# A Comparative Molecular Dynamics Study of Methylation State Specificity of JMJD2A

**DOI:** 10.1371/journal.pone.0024664

**Published:** 2011-09-13

**Authors:** Ozlem Ulucan, Ozlem Keskin, Burak Erman, Attila Gursoy

**Affiliations:** Center for Computational Biology and Bioinformatics and College of Engineering, Koc University, Istanbul, Turkey; Vanderbilt University Medical Center, United States of America

## Abstract

Histone modifications have great importance in epigenetic regulation. JMJD2A is a histone demethylase which is selective for di- and trimethyl forms of residues Lys9 and Lys36 of Histone 3 tail (H3K9 and H3K36). We present a molecular dynamics simulations of mono-, di- and trimethylated histone tails in complex with JMJD2A catalytic domain to gain insight into how JMJD2A discriminates between the methylation states of H3K9. The methyl groups are located at specific distances and orientations with respect to Fe(II) in methylammonium binding pocket. For the trimethyllysine the mechanism which provides the effectual orientation of methyl groups is the symmetry, whereas for the dimethyllysine case the determining factors are the interactions between methyllysine head and its environment and subsequently the restriction on angular motion. The occurrence frequency of methyl groups in a certain proximity of Fe(II) comes out as the explanation of the enzyme activity difference on di- and tri-methylated peptides. Energy analysis suggests that recognition is mostly driven by van der Waals and followed by Coulombic interactions in the enzyme-substrate interface. The number (mono, di or tri) and orientations of methyl groups and water molecules significantly affect the extent of van der Waals interaction strengths. Hydrogen bonding analysis suggests that the interaction between JMJD2A and its substrates mainly comes from main chain-side chain interactions. Binding free energy analysis points out Arg8 as an important residue forming an intra-substrate hydrogen bond with tri and dimethylated Lys9 of the H3 chain. Our study provides new insights into how JMJD2A discriminates between its substrates from both a structural and dynamical point of view.

## Introduction

Histone tails that protrude from the nucleosome are subject to a large number of modifications that include methylation, acetylation, ubiquitilation and phosphorylation. Lysine residues (Lys4, Lys9, Lys27 and Lys36) on histone 3 (H3) tail can be mono-, di- and trimethylated. These differentially methylated residues serve as docking sites for diverse effector proteins, which function in various physiological responses [1]. JMJD2A is a histone demethylase that specifically demethylates Lys9 and Lys36 trimethyl marks on H3 tail. The whole protein consists of 1064 amino acids, which form six separate domains: one Jumonji N (JMJN) domain, one Jumonji C (JMJC) domain, two plant homeodomains (PHD) and two tudor domains. The catalytic-core domain of JMJD2A enzyme consists of the first 350 amino acids which covers JMJN and JMJC domains at the same time [2]. The crystal structure of the catalytic-core domain was first determined by Chen et al. in the presence of Fe(II) with and without α-ketoglutarate [2]. Thereafter, various crystal structures of JMJD2A catalytic-core domain in the presence of distinct substrates came one after another. Chen et al. determined the structure of the catalytic-core in complex with methylated H3 Lys36 (H3K36) peptide substrates [3]. In their study, they mainly addressed the sequence specificity of the enzyme. Additionally they found out that the interactions between enzyme and substrate peptide were mainly main chain-main chain interactions. They also assessed the detailed interactions between methyllysine head and its binding environment. Their claim was that space and the electrostatic environment of the catalytic center affected the specificity for a certain methyl group and allowed for its proper orientation towards the Fe(II) ion.

**Figure 1:**
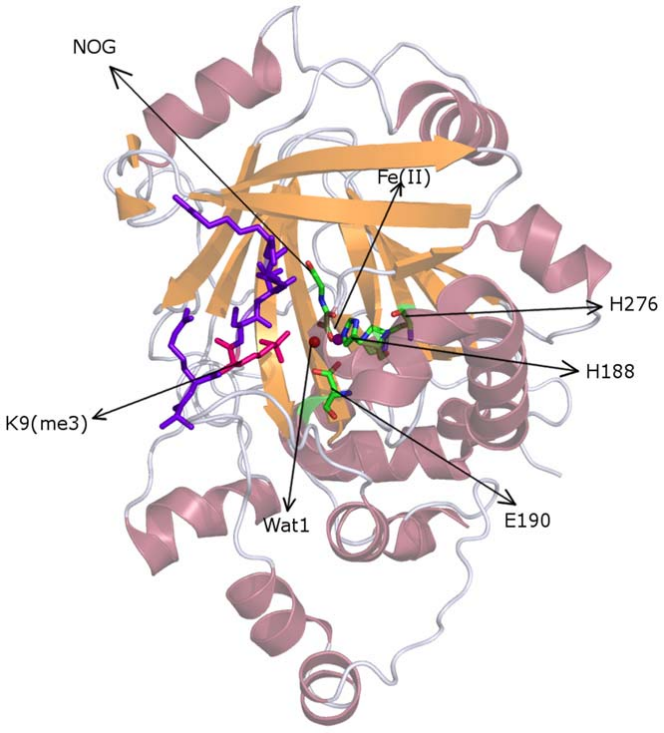
Structure of JMJD2A-H3K9(me3) complex. The enzyme is shown as transparent cartoon in order to expose the deeply buried active site. The H3 tail is shown in violet stick representation highlighting the trimethyllysine with hot pink. The Fe(II) and its coordinating water molecule are shown as purple and red spheres respectively. The residues which coordinate Fe(II) are shown in stick representation indicating the constituent element in color code (C:green, N:nitrogen, O:red).

Ng et al. probed how JMJD2A discriminates between the methylation states and achieves sequence specificity via resolving JMJD2A catalytic domain in complex with mono-, di- and trimethyl forms of H3K9 and trimethyl form of H3K36 [4]. They proposed a mechanism to explain how JMJD2A achieves methylation state selectivity involving contribution of water molecules. Right after that, Couture et al. reported the crystal structure of JMJD2A catalytic-core domain in complex with mono-, di- and trimethylated forms of H3K9 peptide [5]. In their work they stated that the network of C-H—O [6] type hydrogen bonds coordinates the trimethyllysine in JMJD2A active site and positions one methyl group into close proximity of Fe(II). See Figure 1 for the global structure of the enzyme and the buried active site.

Histone modifications have been of great importance due to their role in chromatin structure and organization. Alterations in these modifications can activate oncogenes and inactivate tumor suppressor genes that ultimately result in uncontrollable proliferation. For instance, H3K9 and H3K27 are associated to inactive heterochromatic regions and have been found aberrantly repressed in cancer [7]. Additionally, histone demethylases including JMJ2A have been found to play a role in prostate cancer progression [7], [8]. Like other epigenetic changes histone modifications are promising drug targets since they can be reversed relatively easily through chemotherapeutic intervention compared to genetic changes [7].

Previously, we performed a molecular dynamics simulation analysis of the recognition of JMJD2A-tudor with different histone tails [9]. Our binding free energy calculations showed that H4K20(me2) and H3K9(me3) peptides have the highest and lowest affinity to JMJD2A-tudor, respectively. In this paper, we investigate the methylation-state specificity of JMJDA by employing full atomistic molecular dynamics (MD) simulations of JMJD2A catalytic domain in complex with H3 tail monomethylated at Lys9 (H3K9(me1)), H3 tail dimethylated at Lys9 (H3K9(me2)) and H3 tail trimethylated at Lys9 (H3K9(me3)). To our knowledge, our work is the only computational study that investigates methylation state specificity of JMJD2A from a dynamical point of view. To understand the origin of state selectivity of the enzyme, positional fluctuations are analyzed, hydrogen bonds are located and some critical distances are measured for the three cases. Our calculations point to the importance of water molecules in specific orientation with respect to the methylammonium head. Analyses of the distances and the steric clashes between methyllysine head and methylammonium binding pocket suggest that three distinct sites exist for each methyl group, one of which aligns with Fe(II) cation. For the monomethyllysine case, the site that is adjacent to Fe(II) cation is almost not visited by the methyl group. For this case, the nonproductive orientation is an outcome of avoidance of steric overlaps and presence of water molecules in critical positions. We also show that similar factors provide a productive orientation for dimethyllysine head. For the trimetllysine case the productive orientation is the outcome of the symmetric methyllysine head which generates the same conformation after rotation. Computational binding free energy analyses reveal that recognition is driven by van der Waals and Columbic interactions. The importance of intra-substrate hydrogen bond, which is formed between the side chain of Arg8 and main chain of Lys9 in stabilizing H3 peptide, comes out as the result of energy calculations.

## Results and Discussion

Molecular dynamics simulations for the complexes; JMJD2A with mono-, di- and trimetylated H3 peptide at Lys9 were performed. During the 18 ns of MD simulations, all three enzyme-substrate trajectories exhibit backbone root-mean-square deviation (rmsd) values below 2 Å indicating their stability (See Figure S1). Backbone fluctuations show minor changes in the last 6 ns and 2 ns of simulations for H3K9(me3) and H3K9(me2) respectively. In order to understand the behavior of bonded substrates we also calculated the RMSD of these peptides and provided the results in the right panel of Figure S1. As seen in this figure H3K9(me3) exhibits higher RMSD values compared to other two peptides.

We analyzed the behavior of the metal center throughout the simulations for H3 tail. For each system we found out that the mean fluctuation of Fe(II) cation is less than 1 Å. We further assessed the structure of the metal center taking H3K9(me3) as the model. During the 18 ns of simulations, the overall structure of the metal center remained almost the same (See Figure S2). It was interesting to look at the interactions between Fe(II) cation and its coordinating water molecule. This crystal water molecule together with equilibrium phase remained bonded to Fe(II) almost for 16 ns which is indicative of a strong interaction. Before dissociation of the crystal water molecule, a second water molecule came and bonded to Fe(II). For a while, the two water molecules coordinated Fe(II) and thereafter the crystal water molecule broke away.

We also examined the behavior of Fe(II)-coordinating water molecules for H3K9(me1) and H3K9(me2) cases and provided the results in Figure S3. For both cases the crystal water molecules remain bonded to Fe(II) throughout the simulations. For H3K9(me2) and H3K9(me1) the mean distances between Fe(II) and the relevant water molecules are 2.15 (±0.09) Å and 2.16 (±0.10) Å, respectively. For our satisfaction we probed the interaction between the Fe(II)-coordinating water and its surrounding for each case. Contrary to our expectation, we did not detect any notable hydrogen bonds between the water molecule and its surrounding atoms. It seems that van der Waals and Columbic interactions between the water and Fe(II) cation anchor the water molecule in close proximity of Fe(II). For the three cases the Fe(II)-coordinating water molecule stands somewhere between Fe(II) and the methylammonium head (See Figure S2). This finding is consistent with the mechanism that is proposed for Fe(II)/α-ketoglutarate-dependent hydroxylases [10]. The site in which the Fe(II)-coordinating water molecule sits is most probably where the oxygen molecule binds and activation starts [4].

### Role of Water Molecules

**Figure 2:**
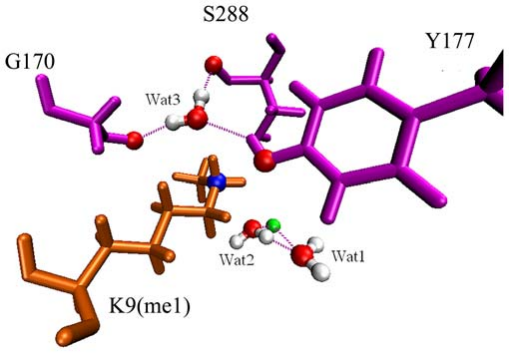
Representative snapshot of the water molecules in certain positions at H3K9(me1) case. Fe(II) cation is shown with green and critical oxygen atoms are shown with red spheres.

Ng and coworkers argued the importance of the water molecules that they detected in certain positions in the crystallographic structure [4]. They demonstrated that in the case of H3K9(me1) and H3K9(me2) the niche of absent methyl groups were occupied by water molecules. It remains unclear if these water molecules are positionally stable and/or they are required for the discrimination of different methyl groups. We examined the behavior of these water molecules and their possible effects on the orientation of methyl groups. For the case of H3K9(me1) two water molecules (Wat2, Wat3) in certain positions were reported and the presence of these water molecules was associated with directing away the single methyl group from the active site [4]. Wat3 remained around its initial position during the first half of the simulation. Within this time course, Wat3 was hydrogen bonded to Glu170, Tyr177 and Ser288 (See Figure 2). Wat2 remained in the close proximity of the methylammonium head throughout the simulation by forming a hydrogen bond with Wat1 and Columbic interaction with Fe(II). Additionally, Wat2 that occupied the location of the absent methyl group in the case of H3K9(me2) remained in the same niche almost throughout the full simulation. To quantify the stability of Wat2 and Wat3 we computed the distance of those molecules to NZ atom of methyllysine and provided the results in A, B and C panels of Figure S4.

### Methylammonium Binding Pocket

**Figure 3:**
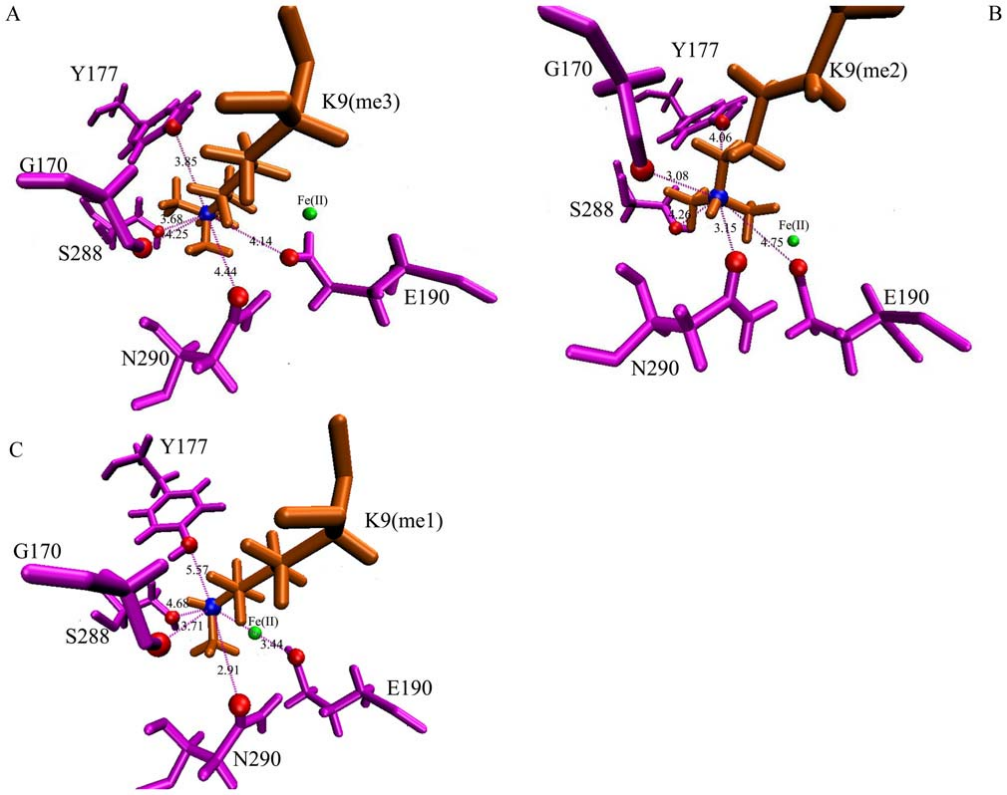
Methylammonium binding pocket.

The NZ of the methyllysine locates in an oxygen-enclosed pocket that is formed by the side chains of Tyr175, Tyr177, Ser288, Asn290 and Glu190 and the backbone oxygen of Gly170. These amino acids together impose the appropriate orientation of the methyllysine side chain in the methylammonium binding pocket [4]. We further assessed this issue, measured the distances between NZ of methyllysine and the surrounding oxygen atoms, and pursued the possible hydrogen and C-H—O bonds. In a previous study Couture et al. reported that the state specificity of JMJD2A results from the network of C-H—O hydrogen bonds in the methylammonium binding pocket [5]. Contrarily, we did not detect any hydrogen bonds in this region. The bond distances were close enough while the angles were not appropriate to form C-H—O type hydrogen bonding [6], [11] despite the loose criteria we used (hydrogen bond distances less than 3.4 Å and donor-hydrogen-acceptor angle greater than 120°). In Figure 3, we provided the mean distances between NZ and the surrounding oxygen atoms for each case. For the trimethyllysine case (See Figure 3, panel *A*) the oxygen atoms locate almost equally apart from NZ but for the dimethyllysine and monomethyllysine that is not the case. For the dimethyllysine case (See Figure 3, panel *B*) the distances between NZ and Gly170 (3.08 Å) and Asn290 (3.15 Å) oxygens become shorter compared to trimethylysine case in which the distances are 4.25 Å and 4.44 Å respectively. In comparison of monomethylysine and other two cases the change of the distance between NZ and Asn290 oxygen (2.91 Å) is prominent (See Figure 3, panel *C*) which is indicative of a strong interaction between these two groups. Moreover, those distances range from 2.91 Å to 5.57 Å and are close enough to form Columbic interactions. The importance of the Columbic interactions in state specificity of JMJD2A was emphasized in a previous study [3] which reported that the distances between NZ of methyllysine and the metylammonium oxygens differs between 3.52 Å and 4.67 Å. However, their results are for H3K36(me3), here we observe similar type of interactions for H3K9(me3). For H3K9(me3) we found out that the distances range between 3.68 Å and 4.44 Å. In this respect, our findings are consistent with experimental results.

**Figure 4:**
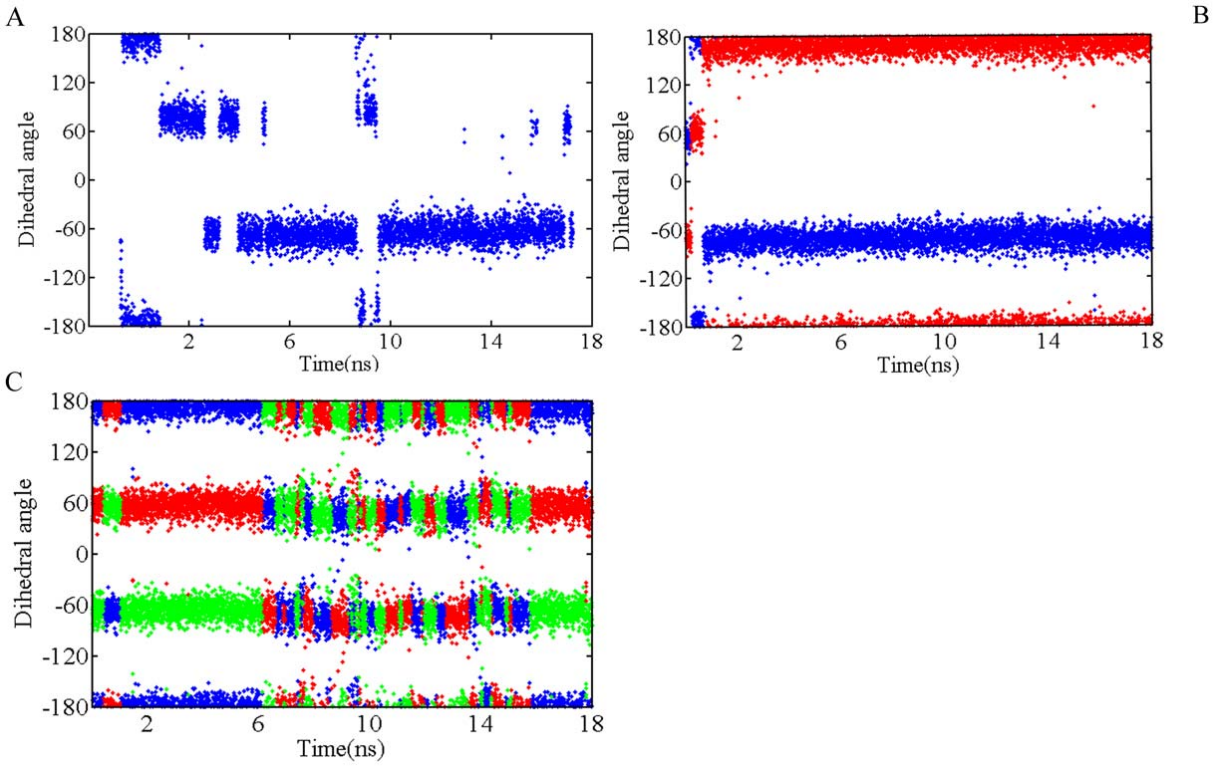
Rotation around CE-NZ bond for H3K9(me1) (panel A), H3K9(me2) (panel B) and H3K9(me3) (panel C). In order to measure the dihedral angle CD, CE, NZ and the methyl carbons (CZ1,CZ2 or CZ3) were used as reference atoms. The resulting angles are color-coded; blue (when CZ1 used), red (when CZ2 used) and green (when CZ3 used). For clarity a value of 4 ps is used for time interval. The angle values are in degree.

To examine the behavior of methylammonium head in the methylammonium binding pocket we measured the torsion rotation around the CE-NZ bond over time, taking CD, CE, NZ and methyllysine head carbons as reference atoms. As seen in panel *C* of Figure 4, trimethyllysine head is almost free whereas dimethylysine (panel *B* of Figure 4) and monomethyllysine (panel *A* of Figure 4) heads are almost completely restricted to rotate around the CE-NZ bond. As can be seen in Figure 4, three discrete torsion states exist for the CE-NZ bond, which forces the NZ-methyl groups to specific orientations in the binding pocket.

Recalling the results obtained for the rotation around the CE-NZ bond and the distance measurements in the methylammonium binding pocket, steric clashes might play a role in the restriction on the angular motions in dimethyllysine and monomethyllysine cases. Since methylammonium has symmetry around the NZ atom of methyllysine, a 120° rotation around the CE-NZ bond results in the same orientation. The equivalence of the occupied volume by each methyl group prevents the atoms from any steric overlaps during rotation. The value of CE-CD-NZ-X (X is the carbon atom of any methyl group) dihedral angle which allows the relevant methyl group to align with Fe(II) is around 180°. For the case of trimethyllysine and dimethyllysine always one methyl group provides the effectual value. The CE-CD-NZ-CZ1 dihedral angle was restricted to around 60 for monomethylysine case. In order to further asses the space problem that arises from steric barriers we picked representative structures, turned the methylammonium head around the CE-NZ bond by −120° each time, and defined the steric clashes. As we expected, when we rotated the trimethylammonium head we did not detect any steric overlaps. But for the dimethyl and monomethyllysine cases we found out several overlaps. For the dimethyllysine case the −120° artificial rotation of the methylammonium head around the bond caused the second methyl group atoms to overlap with Asn290(OD1) and Gly170(O) atoms (See Figure S5 in, panel *A2*). Another −120° artificial rotation created the similar overlaps with the first methyl group atoms (See Figure S5 panel *B2*). The −120° rotation which set the dihedral angle value to ∼180° resulted in overlaps of single methyl group atoms and Asn290(OD1,HD2) and Glu190(OE1) (See Figure S5, panel *A3*). Another −120° rotation around methylammonium head caused the Glu190(OE1,OE2) and Asn290(OD1) to overlap with the methyl group atoms (See Figure S5 panel *B3*).

**Figure 5:**
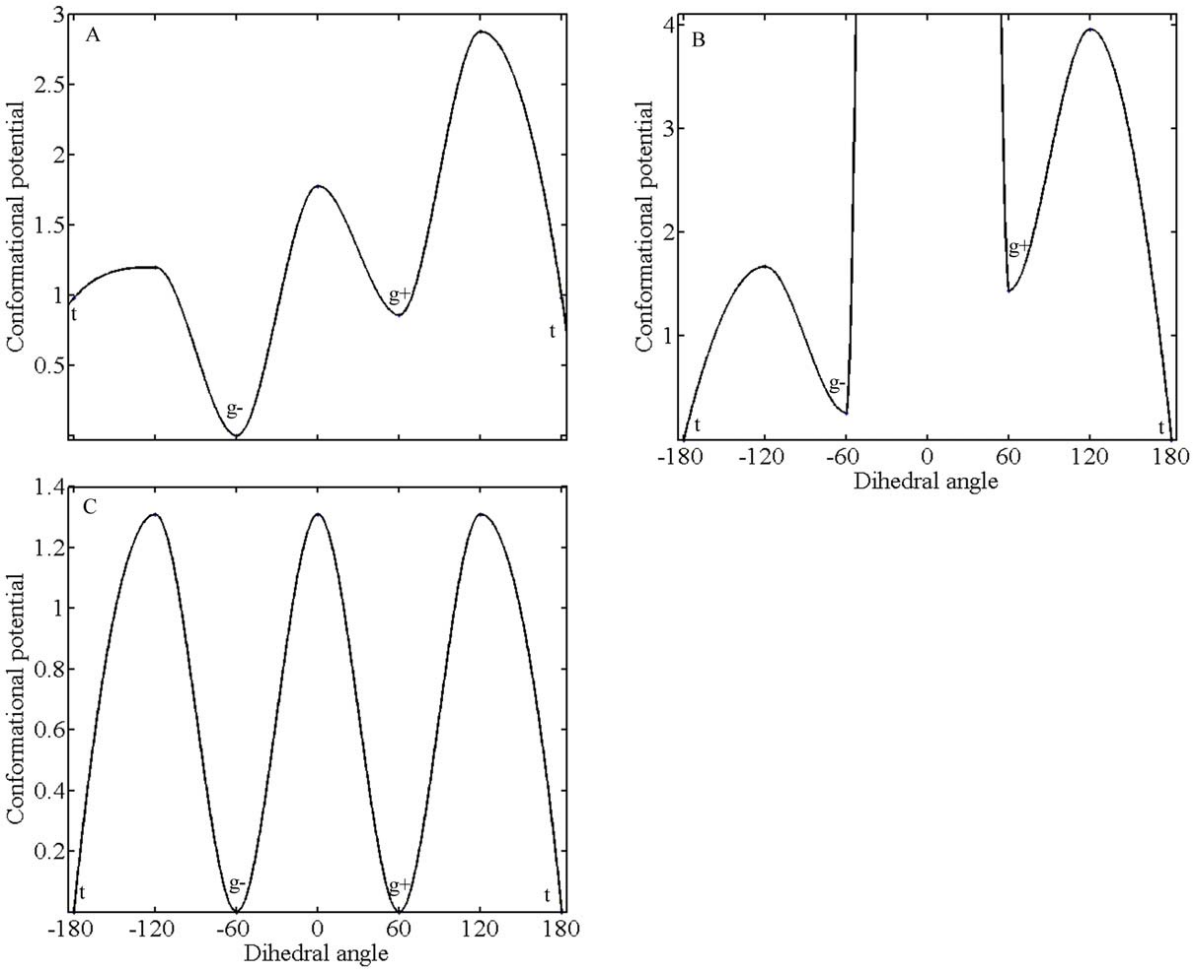
The energy barriers between the three most populated cases for the systems; H3K9(me1) (Panel A), H3K9(me2) (Panel B) and H3K9(me3) (Panel C). The range (−180°)–(180°) was discretized with an interval of 120° to define the states. From the transition rates the energy barriers were calculated as explained in the Methods Section.

In order to compare energies of different rotation states of the methyl groups, we determined the occurrences of these states and obtained relative energy values as explained in Materials and Methods section. We computed the occurrence of each state by utilizing the last 14 ns of the trajectory. We used this part of trajectories for two reasons. First, the bound form of the trimethllysine substrate came to equilibrium in the 4th ns of the trajectory. (See Figure S1, *right* panel). Second, this part of the trajectory makes the probability of occurrence of each methyl group of the trimethyllysine close to each other, as expected. The states were obtained by dividing the range (−180°)–(180°) into three sub ranges where the data highly populated. As seen in Figure 5 the trimethylysine head has three minima, which correspond to the torsion states. The defined torsion states of methyllysine head mostly oscillate at the gauche+ (g+) states around 60 degrees, at the gauche− (g−) states around −60 degrees and at the trans (t) states around 180 degrees (See Figure 5.). Since the energy barriers have equal values, each state is equally visited by the trimethyllysine head. As we see in the Figure 5 monomethyllysine and dimethyllysine cases also have three minima however the energy barriers show variety. For monomethyllysine case one minimum that coincides with (g−) is quite lower, compared to other two. Additionally the transition from state (g+) to state (t) is energetically highly demanding and rare-occurring. For the case of dimethylysine, the energy barrier between (g−) and (g+) almost infinitive and prevents the methyllysine head from a circular motion. The energy barrier between the states (g+) and (t) are higher than its conjugates at mono- and trimethyllysine cases. Most probably, the reason, which impairs the symmetry between the barriers for the case of di- and monomethyllysine, is the steric overlaps that we have already mentioned. During the rotation, the side chains of the residues, which cause overlaps with the methyl groups, must not find a more favorable location. Displacements of the side chains increase the amount of required energy to rotate the methyl ammonium head. Reconsidering the Figure 4 and Figure 5, the most prominent consequence which is common for di- and tri-methyllysine systems and different for monometllysine case is the visitation of trans state. As we indicated before, the state which provide the effectual orientation with respect to Fe(II) is the trans state. However the mechanisms derive the effectual orientation is different for di- and trimethyllysine cases, the outcomes are the same. For the trimethyllysine the mechanism which provides the productive orientation is the symmetry of methyl groups, whereas for the dimethyllysine case the mechanism is the interactions between methyllysine head and its environment and subsequently the restriction on angular motion.

Besides the orientation of the methyl groups relative to Fe(II), the distance between the methyl groups and Fe(II) may be an explanation of the activity specificity of JMJD2A. In order to assess this subject we determined a sphere surrounding Fe(II) and quantified the occurrence of each methyl carbon inside the sphere. We obtained the same trend for different values of the sphere radius (4.6 Å–4.8 Å). Here, we present the results for the value of 4.7 Å. The frequency of finding each of the three methyl carbon of the trimethylammonium head within the 4.7 Å distance to Fe(II) was approximately equal. We observed a significant difference between the H3K9(me3) case and other two cases for finding a methyl carbon closer than 4.7 to Fe(II) (see Table 1). The frequencies of finding a methyl carbon closer than 4.7 Å to Fe(II) are 1.5%, 4.5% and 86% for monomethyllysine, dimethyllysine and trimethyllysine cases respectively. The frequency of finding the methyl groups within a 4.7 Å of Fe(II) can be an explanation for the difference of enzyme activity on tri- and di-methylated substrates. For H3K9(me3), both the symmetry of the three methyl groups and the absence of water molecules raise the probability of finding a methyl group within a 4.7 Å distance of Fe(II).

**Table 1:** The frequency of occurrence of methyl groups within 4.7 Å-proximity of Fe(II).

Systems	CZ1*(%)	CZ2†(%)	CZ3‡(%)	TOTAL(%)
H3K9(me3)	32.0	31.0	23.0	86.0
H3K9(me2)	4.5	0	-§	4.5
H3K9(me1)	1.5	-§	-§	1.5

*, † and ‡; methyl carbons of first, second and third methyl groups, respectively.

§ The system does not have these groups.

### Hydrogen Bonds Analyses

Chen et al. [3] reported that the entire interactions between JMJD2A and its substrates involve 10 hydrogen bonds and one salt bridge. However, those results come from analyses of the crystal structures of trimethylated peptides in complex with JMJD2A. We questioned whether there is any difference between H3K9(me1) H3K9(me2) and H3K9(me3) in hydrogen bonding at the enzyme-ligand interface. In order to select hydrogen bonds we used the criteria hydrogen-bond distance (R), R<3.00 Å and hydrogen-bond angle (θ), 120<θ<180. We computed hydrogen bonds for the first 6 ns of simulations and provided the results in Table S1.

Hydrogen bonding analysis suggests that the interactions between JMJD2A and its substrates mainly involve main chain - side chain interactions. For the trimethylysine case, the prominent eight hydrogen bonds between the enzyme and the substrate involve two main chain-main chain, five main chain-side chain and one side chain-side chain interactions (See Table S1). Only Thr11 forms side chain-side chain interactions with Asp135 of JMJD2A. Lys9 forms main chain – main chain hydrogen bond with Glu169 of the enzyme and main chain – side chain hydrogen bond with Arg8. Backbone atoms of the other residues except Gly12 of the substrates participate in hydrogen bonding with JMJD2A residues. Asn80, Asp 135, Glu169 and Lys235 of JMJD2A are common in hydrogen bond formation for the three systems. The hydrogen bond between Asp311 of enzyme and Arg8 of substrate was observed for H3K9(me2) and H3K9(me3) but not for H3K9(me1).

### Binding Free Energy Calculations

Whetstine et al reported [12] that JMJD2A is a K(me3) specific enzyme, only having activity on K(me2) in the presence of excessive amount of enzyme, and has no activity on monomethyllysine. In accordance with their findings Couture et al showed 20-fold reduced activity of this enzyme on dimethyllysine compared to trimethyllysine [5]. Based on these findings we thought that the activity specificity of JMJD2A might be related to binding affinity of the enzyme to its substrates. To determine whether a relation between activity specificity and binding affinity exists, we computed the binding free energy for the three cases.

We computed the free energies using the MM-PBSA method that we explained in details in the Methods section. In order to calculate the binding free energy we extracted snapshots from 4 ns–16 ns part of each trajectory due to the stability of H9K9(me3) tail in this part (See Figure S1, *right* panel). We determined the time interval of two tandem structures according to time correlation function of total energy. For each case, we found the correlation time to be about 3 ps. However, based on a previous study [13], which emphasized the presence of motional correlations on such small time scales, we extracted the structures with a time interval of 12 ps resulting in 1000 snapshots. To be consistent with the force field, we used a value of 4 for internal dielectric constant.

We provided the binding free energies results in Table 2. We found out −15.41 kcal/mol, −17.77 kcal/mol and −21.04 kcal/mol for monomethyllysine, dimethyllyisme and trimethyllysine respectively. Activity of enzymes needs appropriate positioning of reactants and the formation of accurate interactions between enzymes and substrates. The state specificity of this enzyme may stem from binding affinity to its substrates. Our binding free energy results are consistent with the catalytic activity of JMJD2A on its substrates. According to this results JMJD2A has maximum affinity to H3K9(me3) and minimum affinity to H3K9(me1).

**Table 2:** Binding free energy calculations results based on MM-PBSA.

Systems	MM-PBSA*	*−T*Δ*S* *	ΔGbinding*
H3K9(me3)	−86.98	65.94	−21.04
H3K9(me2)	−82.05	64.28	−17.77
H3K9(me1)	−74.52	59.11	−15.41

*The values are in kcal/mol.

### Binding Free Energy Decomposition

Binding free energy decomposition is a useful method, to determine the contribution of each residue of both binding partners. This method provides insight into the origin of binding. For detailed information on the Binding Free Energy Decomposition Method see Text S1. Since we aim to reveal state specificity of JMJD2A this method may help to determine the interaction types, which anchor the peptides in substrate binding site. We perform binding free energy decomposition calculations for each case with 1000 structures that cover 4–16 ns part of trajectory as well as MM-PBSA calculations. We tabulate results in Table S2 for enzyme residues and Table S3 for substrate residues.

Binding free energy decomposition analyses revealed that Asn86, Asp135, Glu169, Gly170, Val171, Tyr175, Tyr 177, Lys241 and Val313 of JMJD2A make important contributions to binding free energy for three cases (see Table S2). Additionally for the case of H3K9(me3) Glu190, Met242, Thr289, Asp290, Arg309, Asp311 and Met312 of JMJD2A make important contribution to binding free energy. Ile168 and Val313 were reported to make van der Waals interactions with N-terminal residues of H3K9(me3) peptide while Asn86, His240, Lys241 and Met242 were found in same type of interactions with C-terminal of the substrate peptide [5]. Concurrently it was found out that Gly170, again Tyr177, Ser288 and Glu190, which formed methylamonium-binding pocket and Tyr175 Tyr177, Asp191 and Asn290 interacted with side chain of trimethyllysine. Our findings substantially correlate with experimental data except Asp191 and Ser288 [3], [5].

All residues of the substrate peptides seem to be important for binding. As expected the most contribution to binding free energy comes from the modified Lys9. Arg8 is found to be the secondly most important residue in binding. The energy contribution of Arg8 on H3 peptide is in good agreement with occurrence of the intra-substrate hydrogen bonding. The energy contribution of Arg8 in dimethyllysine is the highest, which coincides with the occurrence of the intra-substrate hydrogen bond. For both residues, especially for Lys9, the large part of favorable energy contribution comes from van der Waals interactions. The electrostatic interactions are secondly important.

### Importance of Intra-substrate H-bonding

**Figure 6:**
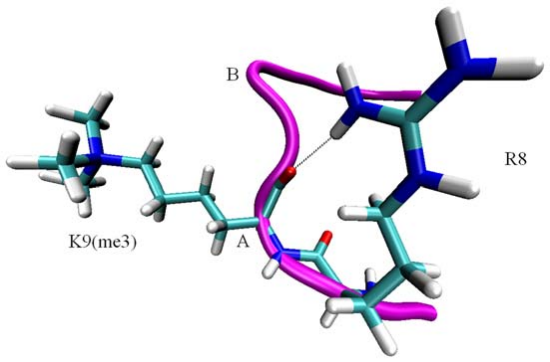
Representative snapshot of H3 Arg8 side chain and H3 Lys (me3) main chain hydrogen bond. The backbone of H3 tail is shown in magenta, the two arcs of W-shaped conformation are indicated with A, and B. Hydrogen bonds are defined by the distance less than 3 Å and donor-hydrogen-acceptor angle greater than 120°.

It was revealed that JMJD2A shows maximal activity with the H3K9(me3) peptide substrate [4], [5], suggesting that this sequence adopts an optimal conformation. The H3K9(me3) substrate gains a broad ‘W’-shaped conformation during binding. This bent peptide conformation is stabilized by intra-substrate hydrogen bond and is required for sequence specificity of JMJD2A [4]. We analyzed all potential hydrogen bond acceptors and hydrogen bond donors. Contrary to expectations, we did not find out a hydrogen bond between H3 Ser10 side chain and main-chain H3 Gly12 that was reported by Ng et al [4]. In their study, they observed a strong reduction in activity of Ser10Ala mutant of H3K9(me3) and related this finding to intra-substrate hydrogen bond. Unexpectedly, we discovered another intra-substrate hydrogen bond which was formed between H3 Arg8 side chain and trimethyllysine main-chain (Figure 6). The side chain of Arg8 is twisted to establish interaction with main chain of trimethylysine. This hydrogen bond occurs in a critical place where the first arc (Shown with A in Figure 6) is formed. The intra-substrate hydrogen bond creates a tensile force, which stabilizes the necessary bent conformation of the substrate peptide, which eventually directs the methyllysine side chain into the methylammonium binding pocket. Throughout the simulation the W-shape of the substrate was conserved which is indicative of stability. This stable conformation is most probably an outcome of the intra-substrate hydrogen bond as well the interactions between enzyme and substrate. Based on this finding we proposed that Arg8 has a critical role in stabilizing the substrate. To further assess the claim we performed computational alanine scanning mutagenesis. We mutated Agr8 to Ala and performed an MD simulation of 12 ns. Comparison of bonded peptide of the wild type and the mutant shows that, the mutant has a much broader ‘W’ shape especially at the point of arc A which implies the significance of the Arg residue at this position.

Additionally, to assess the issue from the energy point of view we calculated the enthalpic component of binding for the mutant system. We found out 2.4 kcal/mol reduction in the enthalpic term compared to the wild type, which indicates the importance of Arg8. Both structural and energetic analyses support the important role of Arg8.

### Conclusion

In this work we have presented the results of our all-atom MD simulations of JMJD2A in complex with its substrates: H3K9(me1), H3K9(me2) and H3K9(me3) to the methylation state specificity of JMJD2A. In total, our simulation time reached 20 ns with 2 ns of equilibration phase for each case. We performed structural, hydrogen bonds, binding free energy, alanine scanning and binding free energy decomposition analyses.

The distances between metal and its coordinating-atoms, during dynamics, seem to be in good agreement with the distances in crystallographic structures. The low values of mean fluctuations of Fe(II) over 18 ns with respect to its initial position are indicative of a successful parameterization.

We examined the behavior of water molecules which were found in certain positions in the crystallographic structure. For H3K9(me1) one water molecule (Wat3) kept its initial position for 10 ns the other molecule (Wat2) remained around its initial positions over 18 ns as well as in the case of H3K9(me2). Wat3 kept its position via forming hydrogen bonds with Ser288 and Gly177 main chain oxygens whereas Wat1 formed hydrogen bonds with Ser288 and Tyr171 side chains.

We examined the behavior of cationic head of modified lysine and the positions of methyl groups with respect to Fe(II) for each case. The methyl groups did not locate randomly and had three rotamer states to occupy. Compared to the H3K9(me3) case, H3K9(me2) and H3K9(me1) cationic heads were much more restricted. We demonstrated that restriction on the angular motion in dimethyllysine and monomethyllysine cases is an outcome of avoidance of steric overlaps. Since methylammonium has symmetry around the NZ atom of methyllysine, 120° rotation of methylammonium head results in an equivalent orientation. The equivalency of the occupied volume by each methyl group prevents the atoms from any steric overlaps during rotation.

The occurrence of methyl groups in a certain proximity of Fe(II) was in good agreement with the enzyme activity difference on its substrates. For H3K9(me3) both the presence of three methyl groups and the absence of water molecules which may restrict orientation of methyl groups raise the probability of occurrence of methyl groups in close proximity of Fe(II). In addition, the increase in atom numbers in the cationic head might strengthen the van der Waals interactions between the head and the iron ion.

Hydrogen bonding analysis suggests that the interaction between JMJD2A and its substrates mainly involves main chains of H3 tail and side chains of the enzyme. 5 of the 8 hydrogen bonds, for the trimethylysine case, form between the main chains of the substrate and the side chains of the enzyme. We found out only 2 main chain-main chain hydrogen bonds between the substrate and the enzyme. This finding contradicts with a previous work which states the majority of the interactions are main chain – main chain interactions [3]. The experimental results come from the system H3K36(me3), which contains a distinct peptide. However, another experimental study on H3K9(me3) emphasizes the importance of backbone hydrogen bond network in recognition [5].

We also performed binding free energy analysis with MM-PBSA method. We carried out MM- GBSA binding free energy decomposition to reveal the critical residues. Our results identified Arg8 as an important residue in two ways. First, it has prominent favorable energy contribution to binding free energy and secondly, it forms intra-substrate hydrogen bond with modified lysine in H3K9(me3) and H3K9(me2). These findings point out the importance of Arg8 and therefore may provide a basis for future experimental studies.

Overall our study provides an insight how JMJD2A discriminates between its substrates from both energetic and structural point of view. Even though a great effort has been made by experimentalist to characterize function and mechanism of JMJD2A, our study is the only computational study that targets JMJD2A from a dynamical point of view. Bringing to light the structural and energetic properties of the enzyme will provide a bases for structure based drug design.

## Materials and Methods

### Preparation of Initial Coordinate Files

The initial coordinates of MD simulations were taken from X-ray structures of the JMJD2A - H3 tail complex. Mono-, di-, trimethylation at Lys9 corresponds to the entries 2OT7 [4] at 2.13 Å, 2OX0 [4] at 1.95 Å and 2OQ6 [4] at 2 Å, respectively in Protein Data Bank (PDB). Although each initial structure contained two copies of the complex, we extracted the B chain of JMJD2A and the conjugate H3 peptide, based on a previous work [2] that reports that JMJD2A functions as a monomer. Since all B chains and the conjugate peptides have the same amino acid sequence, we did not further process the complexes. All histidine residues that do not contribute to coordination of Fe(II) were set to neutral and were protonated at *N_ε_*. The protonation states of His 240 and His188 that coordinate the cation were determined following a previous publication [14] which states that all mononuclear Fe ions adopt *N_ε_*-tautomeric conformation of histidines. All crystallographically resolved water molecules were retained in the systems. Each system was then solvated using TIP3P [15] water in a cubic box with at least 10 Å distances around the solute. The Amber03 [16] force field was used with tLeap in Amber10 [17] for system set up.

### Parameterization of Non-standard Residues

In our systems there exist three non-standard residues (mono-, di- and trimethylated lysine) and the cofactor of the enzyme JMJD2A, *N*-oxalylglycine (NOG). For consistency, we obeyed the protocol proposed by Duan et al. [16] to derive the atomic charges of non-standard residues. (See Text S1.) Equilibrium values of the bond lengths, angles and dihedrals of the cofactor were taken directly from the optimized structure. The force constants of missing parameters were adopted from General Amber Force Field (GAFF) [18] using analogy.

### Parameterization of Metal Centers

JMJD2A histone demethylase has a catalytic motif that includes Fe(II) and a zinc finger motif. In catalytic site Fe(II) is coordinated by two histidine residues (His188 and His276), one glutamic acid (Glu190), one water molecule and the cofactor NOG whereas in coordination of Zn(II) three cystein residues (Cys234, Cys306 and Cys308) and one histidine residue (His240) have a role [2]. Both Fe(II) and Zn(II) belong to the transition metals which are strongly interacting with surrounding molecules and causing large electronic rearrangements [19]. Different schemes for reproducing the parameters of the metal centers and the charge transfer between metal and its coordination sphere have been proposed [19]. For our purpose the most convenient and applicable one was the non-bonded model, which was proposed by Dal Pararo et al [19]. We employed this model for our case with some minor changes. (See Text S1.)

### Simulation Details

All simulations were performed with the molecular dynamics package NAMD 2.6 [20] together with the Duan et al. force field [16]. The systems were energy minimized for 10000 steps. Each system was annealed from 10 to 310 K over a period of 60 ps. The systems were then equilibrated at 310 K using Langevin thermostat with a coefficient of 5/ps in the isobaric-isothermal (NPT) ensemble for 2 ns [21]. Periodic boundary conditions and the hybrid Nose-Hoover Langevin piston method [22], [23] were used to control pressure at 1 atm. After equilibration, dynamics were continued with the same conditions that were used for equilibration for additional 18 ns. All hydrogen bond lengths were constrained with the SETTLE [24] algorithm. A multiple time-stepping algorithm was used, where bonded and the short-range non-bonded interactions were evaluated at every time step and the long-range electrostatic interactions were evaluated at every 2 time steps. A value of 2 ps was used for time step. In order to efficiently treat the electrostatic interactions PME was employed. To treat the short-range interactions a spherical cutoff of 10 Å was used.

### Binding Free Energy-The MM/PBSA Approach

MM-PBSA [25] is a commonly used method to calculate binding free energies. The binding free energy may be calculated by comparison of the trajectory of the complex with separate trajectories of the receptor and the ligand or more frequently from a single trajectory of the complex. In our study we used the single trajectory method [25]. (See Text S1.) Additionally, to reveal the energy contribution of each residue, we performed binding free energy decomposition based on MM-GBSA [26].

### Entropy Calculations

Translational and rotational entropies were calculated from their gas phase partition functions; and vibrational entropy was calculated utilizing normal mode analysis. (See Text S1.) Due to the high computational demand, entropy calculations were performed only for a few snapshots, which might cause a sampling problem. To overcome this problem, we clustered the snapshots according to their similarities and obtained a representative structure for each cluster [27]. Then, we minimized each representative structure in the gas phase using the conjugate gradient method until the root-mean-square of the elements of the gradient vector is less than 10^−3^ kcal mol^−1^ A^−1^. For each minimized snapshot the entropy contributions of each component were computed at 310 K using the NMODE module of Amber8 [28]. After all, the entropy contribution of each cluster to the final entropy was obtained via weighing the relevant entropy value with the occurrence of the cluster. For details see Text S1.

## Supporting Information

Figure S1 **Left Panel**: Backbone RMSD of the Enzyme-substrate complexes during the MD production stage. **Right Panel**: Backbone RMSD of the bonded substrates throughout the MD simulations. For clarity the data have been plotted with a time interval of 4 ps.
(TIF)

Figure S2 **Coordination of Fe(II) in active site. Fe(II) is penta-coordinated by NE(His188), NE(His276), OE1(Glu190), one water molecule(Wat1), O2(NOG) and O2'(NOG). Fe(II) cation is shown by light green sphere, trimethylated Lys9 side chain is shown by magenta.** Other amino acids and the cofactor NOG are shown in licorice representation with the atom type color code (O: Red, N: Blue, C: Green and H: White). All distances are in Å and average over 18 ns of production run.
(TIF)

Figure S3 **The distance of Fe(II)-coordinating water molecules to Fe(II).** **Left panel**: The change in Fe(II)-Wat1 distance throughout 18 ns of MD simulation for H3K9(me2) case. **Right panel**: Fe(II)-Wat1 distance change during 18 ns of production simulation for H3K9(me1). For clarity the data have been plotted with a time interval of 4 ps.
(TIF)

Figure S4 **The Distance of Critical Water Molecules to NZ(Lys9) for H3K9(me1) (panel A :Wat2 and panel B :Wat3) and H3K9(me2) (panel C). For clarity a value of 4 ps is used for time interval.** All distances are in Å.
(TIF)

Figure S5 **Column A**; Orientation of methyllysine head at the CD-CE-NZ-CZ1 dihedral angle value 175°. **Column B**; CD-CE-NZ-CZ1 dihedral angle value 50°. **Colum C**; CD-CE-NZ-CZ1 dihedral angle value −70°. From up to down: H3K9(me3), H3K9(me2), H3K9(me1).
(TIF)

Table S1 **Hydrogen bonds with respective occupancies, distances and deviations from linearity.**
(DOC)

Table S2 **JMJD2A residues which are important in binding.**
(DOC)

Table S3 **Substrate residues which are important in binding.**
(DOC)

Text S1 **Methods.**
(DOC)
