# Supplementary figures and images for: A Comparative Molecular Dynamics Study of Methylation State Specificity of JMJD2A
Source: PLoS One. 2011 Sep 13;6(9):e24664. doi: 10.1371/journal.pone.0024664 (PMC3172282; doi:10.1371/journal.pone.0024664)

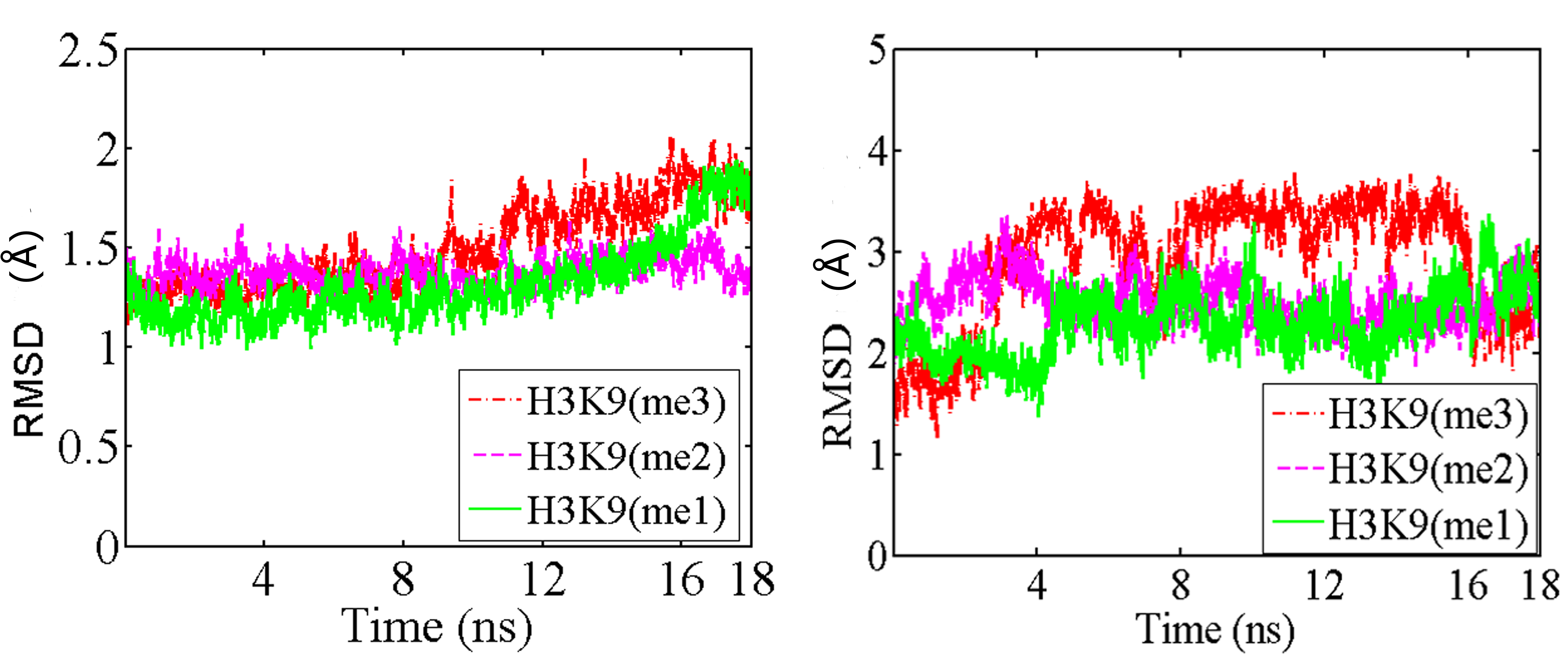

Supplement: Figure S1 — Left Panel: Backbone RMSD of the Enzyme-substrate complexes during the MD production stage. Right Panel: Backbone RMSD of the bonded substrates throughout the MD simulations. For clarity the data have been plotted with a time interval of 4 ps. (TIF) [file pone.0024664.s001.tif]

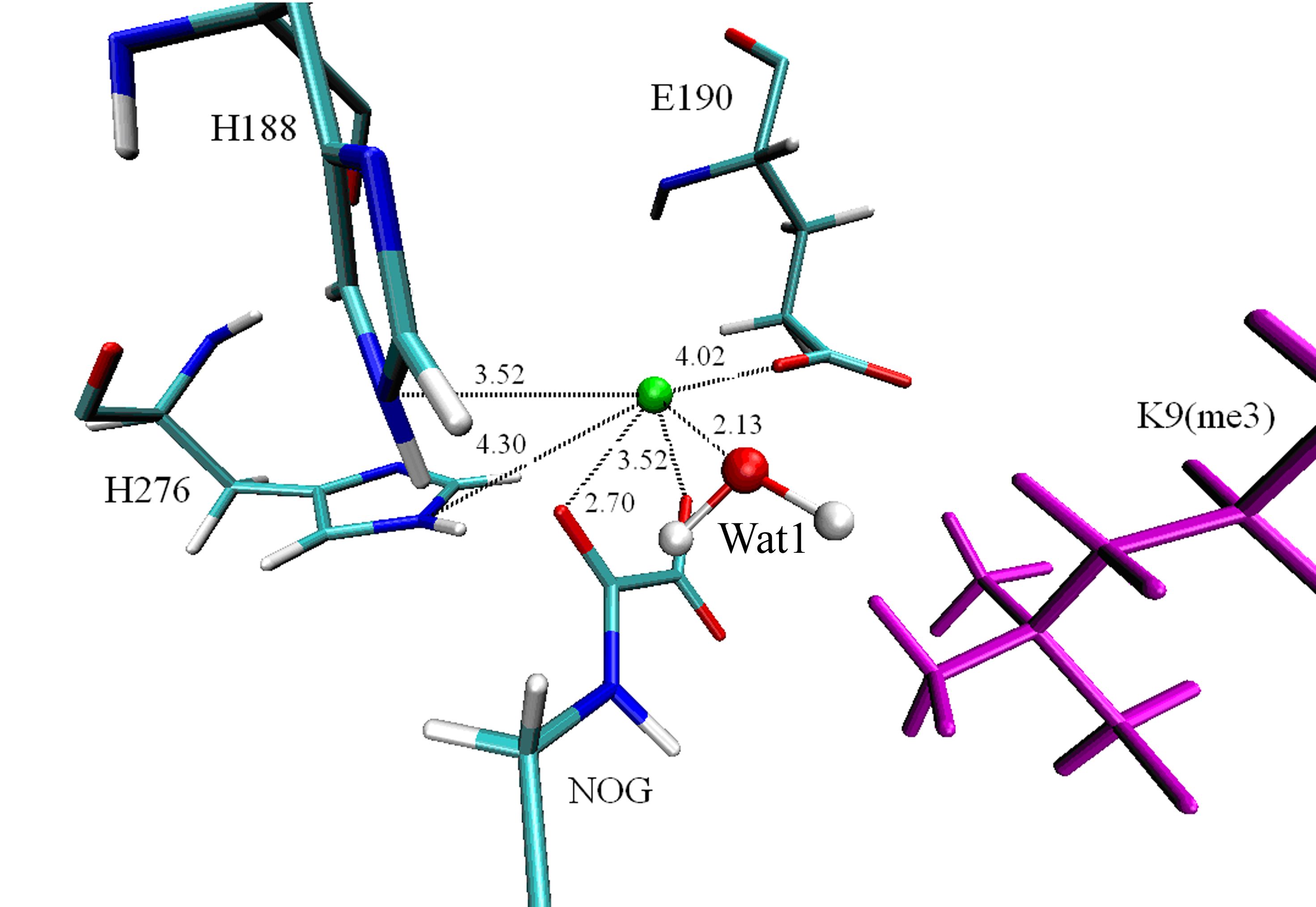

Supplement: Figure S2 — Coordination of Fe(II) in active site. Fe(II) is penta-coordinated by NE(His188), NE(His276), OE1(Glu190), one water molecule(Wat1), O2(NOG) and O2'(NOG). Fe(II) cation is shown by light green sphere, trimethylated Lys9 side chain is shown by magenta. Other amino acids and the cofactor NOG are shown in licorice representation with the atom type color code (O: Red, N: Blue, C: Green and H: White). All distances are in Å and average over 18 ns of production run. (TIF) [file pone.0024664.s002.tif]

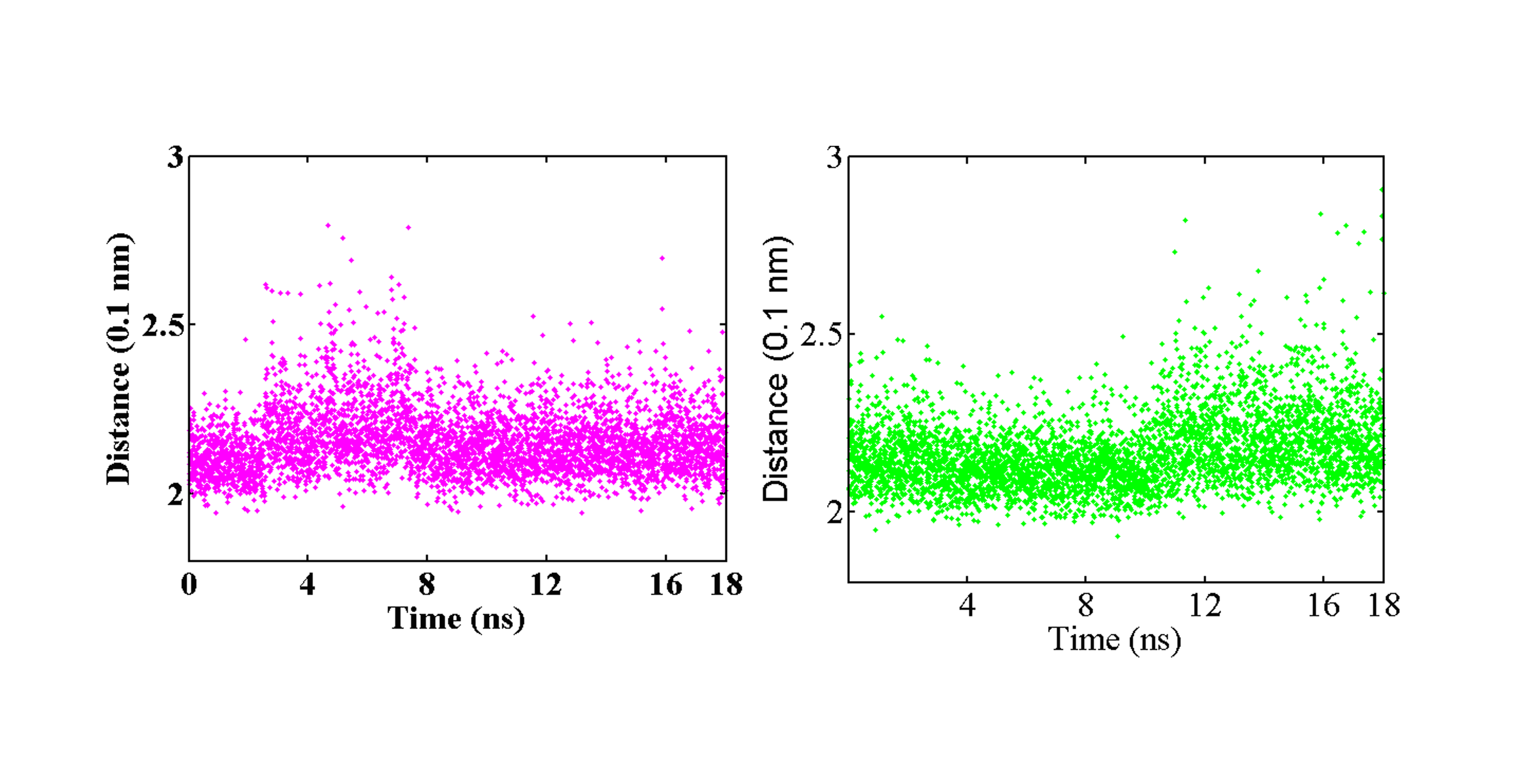

Supplement: Figure S3 — The distance of Fe(II)-coordinating water molecules to Fe(II). Left panel: The change in Fe(II)-Wat1 distance throughout 18 ns of MD simulation for H3K9(me2) case. Right panel: Fe(II)-Wat1 distance change during 18 ns of production simulation for H3K9(me1). For clarity the data have been plotted with a time interval of 4 ps. (TIF) [file pone.0024664.s003.tif]

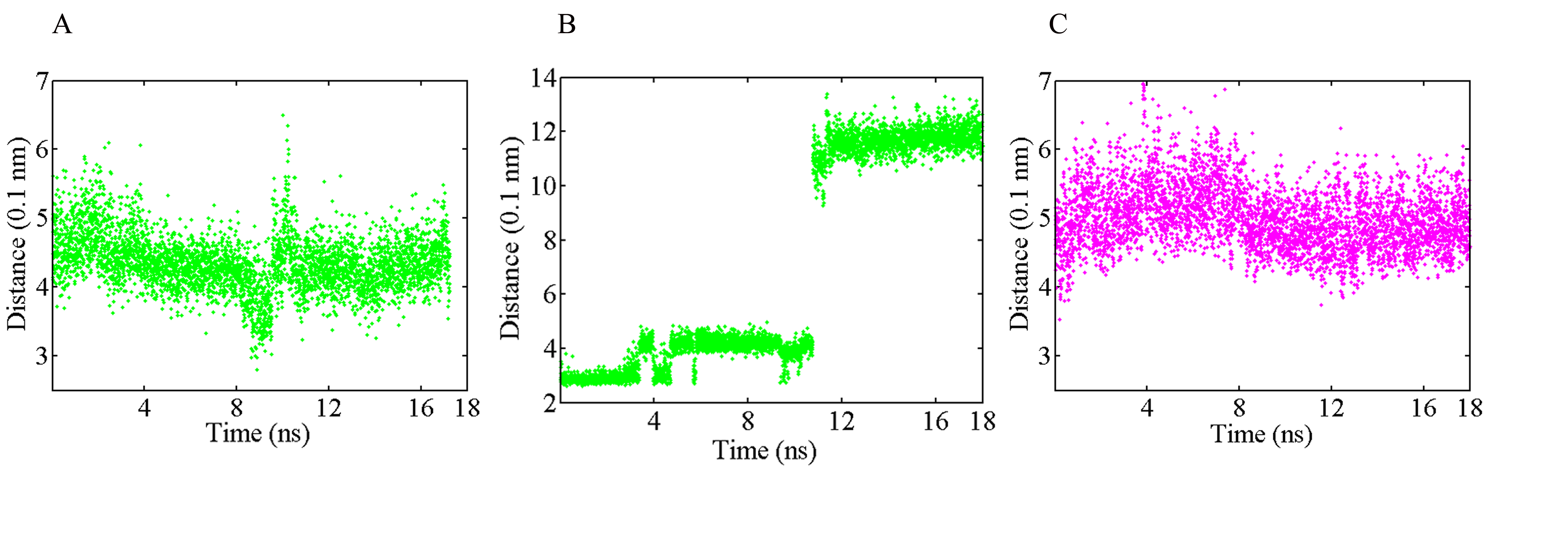

Supplement: Figure S4 — The Distance of Critical Water Molecules to NZ(Lys9) for H3K9(me1) (panel A :Wat2 and panel B :Wat3) and H3K9(me2) (panel C). For clarity a value of 4 ps is used for time interval. All distances are in Å. (TIF) [file pone.0024664.s004.tif]

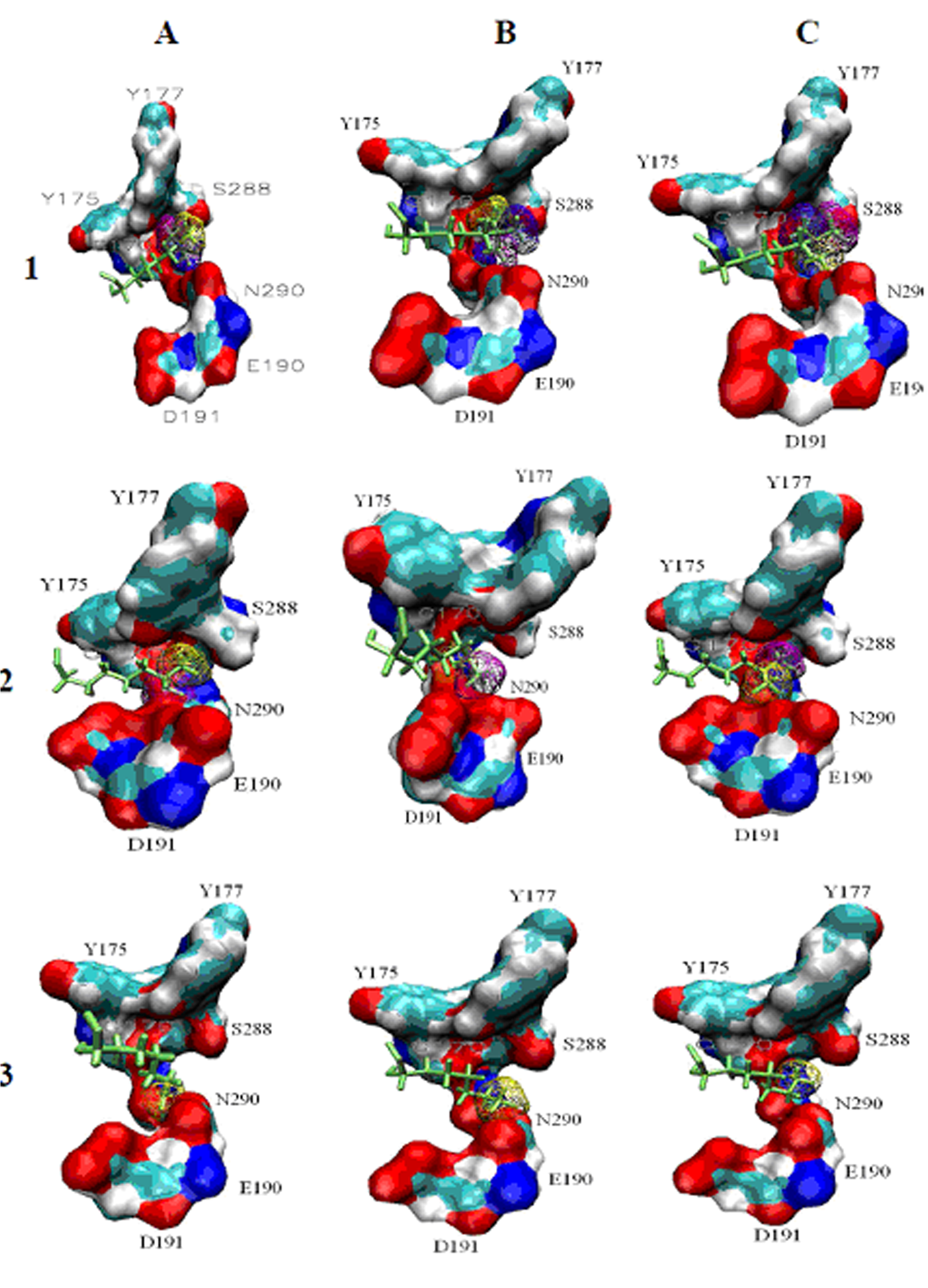

Supplement: Figure S5 — Column A; Orientation of methyllysine head at the CD-CE-NZ-CZ1 dihedral angle value 175°. Column B; CD-CE-NZ-CZ1 dihedral angle value 50°. Colum C; CD-CE-NZ-CZ1 dihedral angle value −70°. From up to down: H3K9(me3), H3K9(me2), H3K9(me1). (TIF) [file pone.0024664.s005.tif]
