# Supplementary material for: A Comparative Molecular Dynamics Study of Methylation State Specificity of JMJD2A
Source: PLoS One. 2011 Sep 13;6(9):e24664. doi: 10.1371/journal.pone.0024664 (PMC3172282; doi:10.1371/journal.pone.0024664)
Supplement: Table S1 — Hydrogen bonds with respective occupancies, distances and deviations from linearity. (DOC) [file pone.0024664.s006.doc]

Table S1: Hydrogen bonds with respective occupancies, distances and deviations from linearity. Hydrogen bonds are defined by the donor-acceptor distance less than 3Å and donor-hydrogen-acceptor angle grater than 120^o^. Only bonds with occupancies higher than 20% are shown. The occupancies are computed from the first 6 ns of production simulations. The receptor residues are shown in bold.

| **SYSTEM** | **Donor** | **Acceptor** | **% Occupancy** | **Distance** | **Angle** |
| --- | --- | --- | --- | --- | --- |
| H3K9(me3) | **Glu169(O)** | Lys9(N) | **56,36** | **2.85 (0.08)** | **24.49 (12.49)** |
| H3K9(me3) | **Asp311(O)** | Arg8(N) | **51,70** | **2.87 (0.08)** | **29.53 (11.62)** |
| H3K9(me3) | **Asp135(OD2)** | Thr11(N) | **46,90** | **2.86 (0.08)** | **22.37 (12.47)** |
| H3K9(me3) | Gly13(O) | **Asn86(ND2)** | **38,37** | **2.88 (0.08)** | **22.14 (11.43)** |
| H3K9(me3) | **Asp135(OD1)** | Thr11(OG1) | **32,45** | **2.73 (0.13)** | **22.94 (12,41)** |
| H3K9(me3) | Lys14(O) | **Arg309(NH2)** | **27,58** | **2.78 (0.10)** | **29.22 (10.56)** |
| H3K9(me3) | **Lys9(O)** | Arg8(NE) | **23,32** | **2.87 (0.08)** | **26.64 (12.11)** |
| H3K9(me3) | Ser10(O) | **Lys241(NZ)** | **21,25** | **2.81 (0.09)** | **29.51 (13.82)** |
| H3K9(me3) | Gly14(O) | **Arg309(NH1)** | **20,45** | **2.81(0,09)** | **31.10 (14.66)** |
| H3K9(me2) | **Asp311(O)** | Arg8(N) | **60,36** | **2.87 (0.08)** | **27.24 (11.60)** |
| H3K9(me2) | Lys9(O) | **Arg8(NE)** | **57,43** | **2.86 (0.08)** | **27.03 (12.11)** |
| H3K9(me2) | Ala7(O) | **Glu169(N)** | **45,70** | **2,88 (0,08)** | **26,96 (11,04)** |
| H3K9(me2) | Gly13(O) | **Asn86(ND2)** | **38,37** | **2.88 (0.08)** | **22.14 (11.43)** |
| H3K9(me2) | Arg8(O) | **Met313(N)** | **35,90** | **2,80 (0,10)** | **35,61 (13,30)** |
| H3K9(me2) | **Glu169(O)** | Lys9(N) | **33,51** | **2.90 (0.07)** | **25.80 (11.96)** |
| H3K9(me2) | **Asp135(OD1)** | Thr11(OG1) | **32,51** | **2.75 (0.12)** | **16.24 (8,85)** |
| H3K9(me2) | Ser10(O) | **Lys241(NZ)** | **29,91** | **2,82 (0,09)** | **25,16 (13,18)** |
| H3K9(me2) | **Asp135(OD2)** | Thr11(N) | **23,92** | **2.86 (0.08)** | **22.37 (12.47)** |
| H3K9(me1) | **Glu169(O)** | Lys9(N) | **76,68** | **2,86 (0.08)** | **19.95 10.11)** |
| H3K9(me1) | **Asp135(OD2)** | Thr11(OG1) | **64,96** | **2,69 (0,11)** | **15,59 (8,09)** |
| H3K9(me1) | Ser10(O) | **Lys241(NZ)** | **58,03** | **2,81 (0,09)** | **21,06 (10,38)** |
| H3K9(me1) | Gly13(O) | **Asn86(ND2)** | **44,24** | **2,88 (0,07)** | **19,07 (9,29)** |
| H3K9(me1) | Ala7(O) | **Glu169(N)** | **35,04** | **2,89 (0,07)** | **30,88 (11,70)** |
| H3K9(me1) | **Asp135(OD1)** | Thr11(OG1) | **33,71** | **2,71 (0,13)** | **23,20 (13,98)** |
| H3K9(me1) | **Asp135(OD2)** | Thr11(N) | **24,92** | **2,85 (0,08)** | **25,20 (14,98)** |
