# Supplementary material for: A Comparative Molecular Dynamics Study of Methylation State Specificity of JMJD2A
Source: PLoS One. 2011 Sep 13;6(9):e24664. doi: 10.1371/journal.pone.0024664 (PMC3172282; doi:10.1371/journal.pone.0024664)
Supplement: Table S2 — JMJD2A residues which are important in binding. (DOC) [file pone.0024664.s007.doc]

Table S2: JMJD2A residues which are important in binding.

| Enzyme  Residues^*^ | H3K9(me1) | H3K9(me2) | H3K9(me3) |
| --- | --- | --- | --- |
| Tyr85 | -1.76 | -^†^ | -^†^ |
| Asn86 | -2.91 | -1.34 | -1.86 |
| Asp135 | -2.14 | -2.29 | -2.31 |
| Ile168 | -1.64 | -1.62 | -^†^ |
| Glu169 | -2.31 | -2.66 | -1.93 |
| Gly170 | -1.18 | -1.21 | -1.31 |
| Val171 | -1.41 | -1.49 | -1.15 |
| Tyr175 | -2.20 | -2.82 | -2.31 |
| Tyr177 | -1.05 | -1.14 | -1.73 |
| Glu190 | -^†^ | -^†^ | -1.43 |
| His240 | -1.41 | -^†^ | -^†^ |
| Lys241 | -2.42 | -1.50 | -2.82 |
| Met242 | -2.07 | -^†^ | -1.07 |
| Thr289 | -^†^ | -^†^ | -1,04 |
| Asn290 | -^†^ | -1,04 | -1.02 |
| Arg309 | -^†^ | -3.43 | -2.80 |
| Asp311 | -^†^ | -2.32 | -3.10 |
| Met312 | -^†^ | -3.56 | -1.07 |
| Val313 | -1.53 | -1.29 | -1.76 |

* Only the residues that make favorable contribution more than 1 kcal/mol are shown.

^†^ The values are less than the threshold.
