# Supplementary material for: A Comparative Molecular Dynamics Study of Methylation State Specificity of JMJD2A
Source: PLoS One. 2011 Sep 13;6(9):e24664. doi: 10.1371/journal.pone.0024664 (PMC3172282; doi:10.1371/journal.pone.0024664)
Supplement: Table S3 — Substrate residues which are important in binding. (DOC) [file pone.0024664.s008.doc]

Table S3: Substrate residues which are important in binding.

| Substrate  Residues^*^ | H3K9(me1) | H3K9(me2) | H3K9(me3) |
| --- | --- | --- | --- |
| Ala7 | -3.40 | -3.67 | -2.89 |
| Arg8 | -4.82 | -8.21 | -5.97 |
| Lys9(me1/2/3) | -7.10 | -12.7 | -13.56 |
| Ser10 | -2.73 | -2.86 | -3.18 |
| Thr11 | -3.65 | -5.07 | -3.92 |
| Gly12 | -2.39 | -2.17 | -3.15 |
| Gly13 | -3.34 | -2.25 | -2.86 |
| Lys14 | -2.59 | -3.43 | -4.58 |

* Only the residues that make favorable contribution more than 1 kcal/mol are shown.
