# Supplementary material for: A Comparative Molecular Dynamics Study of Methylation State Specificity of JMJD2A
Source: PLoS One. 2011 Sep 13;6(9):e24664. doi: 10.1371/journal.pone.0024664 (PMC3172282; doi:10.1371/journal.pone.0024664)
Supplement: Text S1 — Methods. (DOC) [file pone.0024664.s009.doc]

**METHODS**

### Parameterization of Non-standard Residues

Each amino acid was characterized by a dipeptide fragment, which comprised the amino acid, N-methyl (Nme) group at N-terminal and acetyl group (Ace) at C-terminal. For each dipeptide, two structures with main-chain dihedral angles (phi, psi) constrained to alpha helical and beta stranded conformations were prepared and subjected to energy minimization by using the program Accelrys Discovery Studio^1^. These dipeptide conformers were then further geometry optimized at the HF/6-31G level of Quantum Mechanics (QM). All QM calculations were done by using the Gaussian simulation package 03^2^. Molecular electrostatic potential for each conformer was calculated by using density functional theory (DFT) method B3LYP with the ccpVTZ basis set. The IEFPCM continuum solvent model was employed to imitate an organic solvent environment (ε= 4). Atomic charges were obtained by fitting the molecular electrostatic potential of dipeptides using the method Restraint Electrostatic Potential (RESP)^3^. The fitting phase consisted of two stages: In the first stage, the conformers of each dipeptide were combined. In the second stage, the atomic charges of equivalent atoms were equated and the charges of terminal groups and heavy atoms were fixed. All fitting calculations were done with the RESP module of Amber10 suit of package. For preparation of Gaussian03 and RESP input files and processing of output files of these programs, the version III of Resp Esp Charge Drive (R.E.D.)^4^ program was used to accelerate the process.

For charge derivation of the cofactor the same protocol with minor changes was used. A single conformation of NOG was prepared for QM calculations. Since the cofactor was a standalone molecule, no blocking groups were added to the termini.

### Parameterization of Metal Centers

For parameterization of metal centers, to avoid any bias the initial structure was taken from the PDB entry 2OQ7^5^ (2.13 Å) which is not one of our initial structures of MD simulations. In the PDB file Ni(II) ion was replaced by a Fe(II) ion whereas the cofactor NOG was kept. Normally at physiological conditions, the α-ketogluterate (α-KG) is found as a cofactor. It was reported by a previous work that NOG occupies the same position and forms the same interactions with α-KG^6^. After the preparation phase the initial structure was subjected to a two stage minimization process by keeping the backbone atoms fixed. For minimization purposes the Sander module of Amber 10 was used. Subsequently, the metal ions with their first coordination shell were extracted from the energy minimized structure. Zn(II) model was prepared for quantum mechanical calculations by cutting the amino acids from their Cβ atoms. For Fe(II) center, together with the side chains of the amino acids, interacting portion of the cofactor was also used. For geometry optimization, the density functional theory method B3LYP with the base 6-31G** was used, constraining the Cβ positions to mimic the protein scaffold. After structure relaxation, a single point calculation was performed using the B3LYP method and IEFPCM continuum solvent model at dielectric constant 4. This step was performed using the ccpVTZ basis set on all atoms except the metals, for which the TVPZ basis set that is commonly used for transition metals was employed. Finally, the RESP method was used to obtain atomic point charges, which reproduce the electrostatic potential, calculated in the previous step.

## Binding Free Energy-The MM/PBSA Approach

The binding free energy of the two molecules (receptor and ligands) is the difference between the free energy of the complex and that of the receptor and the ligand;

|  | **( )** |
| --- | --- |

where G is the Gibbs free energy, and the subscripts identify the systems. The overbars here and in the following equations denote the time average taken over the full trajectory. According to this method the free energy of each species is calculated as

|  | **( )** |
| --- | --- |

Here $G_{molecule}$ and $E_{MM}$denote the computed average free energy and average molecular mechanical energy,

|  | **( )** |
| --- | --- |

where the indices on the right hand side correspond to bond, angle, torsion, van der Waals and electrostatic terms in the molecular mechanical force field. $\bar{G}_{PBSA}$ is the solvation free energy. This term has two parts.

| $\bar{G}_{PBSA}=G_{elect}+G_{non-elect}$ | **( )** |
| --- | --- |

The First part ($\text{G}_{\text{elect}}$) is the polar part and comes from a numerical solution of the Poisson-Boltzmann equation. The second part ($G_{non-elect}$) is the cost of opening a cavity in the condensed phase and is generally estimated as the product of the surface area and an effective surface tension term^7^:

| $\bar{G}_{non-elect}=\gamma SASA+b$ | **( )** |
| --- | --- |

Here, $\gamma$ is the surface tension parameter set to 0.0072, $SASA$ is the solvent accessible surface area of molecule of interest determined by the ICOSA^8^ method and b is a parameterized value, set to 0.0 for both PB and GB in this study.

**Binding Free Energy Decomposition**

Decomposing $\Delta G$ in terms of contributions from residues of both binding partners provides insight into origin of binding on an atomic level. Based on this, Golkhe and coworkers[9] proposed a scheme which enables us to investigate the contribution of each residue to the binding free energy by means of component analysis. Reconsidering equation (1) in this respect we reproduce:

| $\begin{matrix} \Delta G=\sum_{\begin{matrix} j\in complex\wedge\\ j\in receptor \end{matrix}} (\left\langle G_{complex}(i,j)\right\rangle-\left\langle G_{receptor}(i,j)\right\rangle) \\ +\sum_{\begin{matrix} j\in complex\wedge\\ j\in ligand \end{matrix}} (\left\langle G_{complex}(i,j)\right\rangle-\left\langle G_{ligand}(i,j)\right\rangle) \\ -H_{trans/rot} \end{matrix}$ | **( 6 )** |
| --- | --- |

Here, j stand for residues either located on receptor or protein and i stand for number of snapshots. There by each addend provide the contribution to binding free energy by residue j averaged over snapshot i. Since the energy contribution due to translational and rotational degrees of freedom is already considered in above equation, $G_{molecule}(i,j)$includes contributions from internal gas-phase energy, solvation free energies and entropies:

$$G_{molecule}(i,j)=H_{molecule}^{gas}(i,j)+G_{molecule}^{solvation}(i,j)-TS_{molecule}(i,j)$$

Considering the fact that the total electrostatic work of creating a given charge distribution within a solute, which buried in a solvent is a quadratic function of charges in the GB formula, a GB analogue of the electrostatic potential can be defined at the $r_{l}$ position of each atom:

| $\Phi(r_{l})=-\sum_{k} (1-\frac{\exp(\kappa f_{kl}^{GB}}{\varepsilon_{w}})\frac{q_{k}}{f_{kl}^{GB}(r_{kl})}+\sum_{k\neq l} \frac{q_{k}}{r_{kl}}$ | **( 7 )** |
| --- | --- |

where $\varepsilon_{w}$is the dielectric constant of the solvent and $q_{k}$are atomic partial charges and *Κ* is Debye-Hückel screening parameter. In equation (7) $f_{kl}^{GB}$is a certain smooth function which is assumed to depend only upon atomic radii and interatomic distances $r_{kl}$[10]. Thus the electrostatic contribution from residue j is obtained according to:

| $\begin{matrix} G_{elec}^{molecule}(i,j)=-\frac{1}{2}\sum_{l\in j} \sum_{k} (1-\frac{\exp(-k)}{\varepsilon_{w}})\frac{q_{k}q_{l}}{f_{kl}^{GB}(r_{kl})} \\ +\frac{1}{2}\sum_{l\in j} \sum_{k\neq l} \frac{q_{k}q_{l}}{r_{kl}} \end{matrix}$ | **( 8 )** |
| --- | --- |

The first term on the right hand side of above equation determines the electrostatic contribution to the free energy of solvation for residue j of snapshot i; whereas the second term stands for the gas-phase charge–charge interactions.

The solvent-accessible surface area per atom was computed with a recursive algorithm[8] that uses icosahedra centered on this respective atom as starting point. In every step each triangular face of the polyhedron is divided into four pieces of equal size, thus a better approximation of a sphere is obtained.

## Entropy Calculations

To understand the energetic determinants of substrate recognition at molecular level we need to consider the enthalpic and entropic components of binding. Enthalpic contributions provide a measure of the strength of interactions between the receptor and the ligand. Entropic contributions involve the change in solvent entropy and the loss of solute conformational degrees of freedom.

Eq. 2 can be rewritten as:

| $\bar{G}_{molecule}=H_{molecule}-TS_{MM}$ | **(9 )** |
| --- | --- |

Where and are the enthalpic and the entropic contributions, respectively.

The entropy term comprises the translational, rotational, vibrational and configurational entropies of the solute and can be expressed as

| $S_{MM}=S_{trans}+S_{rot}+S_{vib}+S_{config}$ | **(10)** |
| --- | --- |

$S_{trans}$ and $S_{rot}$ are the entropic contributions from translational and rotational motions, respectively.

Entropy calculations were performed by NMODE module of Amber8. NMODE calculates the $S_{trans}$and $S_{rot}$ from their gas phase partition functions and $S_{vib}$from vibrational motion. In this study we utilized normal mode analysis to obtain the vibrational entropy.

|  |  |
| --- | --- |

$S_{config}$ is the configurational entropy from the side chains reorganization effects which was ignored in this study.

The input for NMODE is snapshots from complex structure and from both binding partners. The larger the number of snapshots is the more accurate the calculations are. Since the entropy calculations by NMODE are computationally very demanding, the calculations can be done only for a few snapshots. The approach that is usually followed in literature is to extract a few equally spaced snapshots and run calculations on them. Instead of just running calculations on equally spaced snapshots we clustered all snapshots based on their structural similarity. Afterwards we performed entropy calculations on the representative snapshot from each cluster and weighted the results by fraction of each cluster. By following the clustering approach one can elevate the accuracy of the calculations.

## Activation Energy Calculations

In order to calculate activation energies of conformational transitions, we utilized the Arrhenius equation:

|  | $k_{t}=Ae^{-\frac{E_{A}}{RT}}$ or $\ln k_{t}=-\frac{E_{A}}{RT}+\frac{c}{RT}$ | **(11)** |
| --- | --- | --- |

In above equation$k_{t}$ , $A$ and $E_{A}$ stand for the transition rate between the states, temperature dependent constant and the activation energy of conformational transition, respectively. Due to we do not have any information about the quantity$A$, we simply equated to some constant c.

**REFERENCES**

1. Studio A. 1.7, Accelrys Software Inc., San Diego, CA, USA. 2006.

2. Frisch M, Trucks G, Schlegel H, Scuseria G, Robb M, Cheeseman J, Montgomery Jr J, Vreven T, Kudin K, Burant J. gaussian 03, Gaussian. Inc, Pittsburgh, PA 2003.

3. Bayly C, Cieplak P, Cornell W, Kollman P. A well-behaved electrostatic potential based method using charge restraints for deriving atomic charges: the RESP model. The Journal of Physical Chemistry 1993; 97(40):10269-10280.

4. Pigache A, Cieplak P, Dupradeau F. Automatic and highly reproducible RESP and ESP charge derivation: application to the development of programs RED and X RED. 2004.

5. Ng S, Kavanagh K, McDonough M, Butler D, Pilka E, Lienard B, Bray J, Savitsky P, Gileadi O, von Delft F. Crystal structures of histone demethylase JMJD2A reveal basis for substrate specificity. Nature 2007; 448(7149):87-91.

6. Chen Z, Zang J, Kappler J, Hong X, Crawford F, Wang Q, Lan F, Jiang C, Whetstine J, Dai S. Structural basis of the recognition of a methylated histone tail by JMJD2A. Proceedings of the National Academy of Sciences 2007; 104(26):10818.

7. Sitkoff D, Sharp K, Honig B. Accurate calculation of hydration free energies using macroscopic solvent models. The Journal of Physical Chemistry 1994; 98(7):1978-1988.

8. Rarey M, Kramer B, Lengauer T, Klebe G. A fast flexible docking method using an incremental construction algorithm. Journal of molecular biology 1996; 261(3):470-489.

9. Holger Gohlke, C.K.a.D.A.C., *Insights into Protein–Protein Binding by Binding Free Energy Calculation and Free Energy Decomposition for the Ras–Raf and Ras–RalGDS Complexes.* J Mol Bio, 2003. **330**: p. 891–913.

10. Onufriev A., Bashford, D., Case, D. A. *Modification of the Generalized Born Model Suitable for Macromolecules*. Vol. 104. 2000.
